# The High‐Pressure Oxide Tb_3_O_5_ and its Non‐Centrosymmetric Low‐Temperature Polymorph–A Comprehensive Study

**DOI:** 10.1002/chem.201804006

**Published:** 2018-10-01

**Authors:** Matthias Glätzle, Oliver Janka, Volodymyr Svitlyk, Dmitry Chernyshov, Manfred Bartsch, Helmut Zacharias, Rainer Pöttgen, Hubert Huppertz

**Affiliations:** ^1^ Institut für Allgemeine Anorganische und Theoretische Chemie Universität Innsbruck Innrain 80–82 6020 Innsbruck Austria; ^2^ Institut für Anorganische und Analytische Chemie Universität Münster Corrensstrasse 30 48149 Münster Germany; ^3^ European Synchrotron Radiation Facility 38000 Grenoble France; ^4^ Center for Soft Nanoscience Universität Münster Busso-Peus-Strasse 10 48149 Münster Germany

**Keywords:** high-pressure chemistry, lanthanides, magnetic properties, solid-state structures, terbium oxide

## Abstract

In this article, the first thoroughly characterized mixed‐valent binary rare earth oxide synthesized under high‐pressure/high‐temperature conditions, and its low‐temperature polymorph are reported. Crystalline HT‐HP‐Tb_3_O_5_ has been prepared from an equimolar mixture of Tb_4_O_7_ and Tb_2_O_3_ under reaction conditions of 8 GPa and 1323 K. Single‐crystal X‐ray structure determination showed that HT‐HP‐Tb_3_O_5_ crystallizes in the orthorhombic space group *Pnma*, isopointal to the β‐Yb_5_Sb_3_‐type structure. Temperature‐dependent measurements of the magnetic susceptibility showed that HT‐HP‐Tb_3_O_5_ is a Curie–Weiss paramagnet. The observed effective magnetic moment of *μ*_eff_=9.21(2) *μ*_B_ per formula unit fits well to the calculated moment of *μ*_calc_=9.17 *μ*_B_. Low‐field measurements revealed antiferromagnetic ordering at *T*_N_=3.6(1) K. Heat capacity measurements indicated an intrinsic structural phase transition of HT‐HP‐Tb_3_O_5_ at low temperature, which was confirmed by synchrotron X‐ray powder diffraction data recorded at 2 K. The metastable high‐pressure modification HT‐HP‐Tb_3_O_5_ undergoes a *translationengleiche* transition from space group *Pnma* to *Pn*2_1_ *a* (non‐standard setting of *Pna*2_1_), leading to the low‐temperature polymorph LT‐HP‐Tb_3_O_5_ by loss of a mirror plane (displacive phase transition).

## 1 Introduction

The most stable rare earth compounds are the oxides, which typically form when firing rare earth metals in the ambient atmosphere.^1^ Due to their electronic configurations, all rare earths (*RE*) are known to exist at least in a trivalent state in various compounds, for example, the well‐known sesquioxides *RE*_2_O_3_.^2^ Five distinct crystalline polymorphs of the rare earth sesquioxides have been identified, designated as *A*‐, *B*‐, *C*‐, *H*‐, and *X*‐type; the latter two only forming above 2273 K. Tb_2_O_3_ is known to exist in the cubic *C*‐Mn_2_O_3_ (bixbyite)‐type, (*Ia* $\bar{3}$ ), related to a doubled fluorite structure with regularly ordered vacancies on 1/4th of the oxygen positions.^3^ At a temperature of 1178 K and a pressure of 2.5 GPa, the monoclinic *B*‐Tb_2_O_3_ (*C*2/*m*) is formed in a reversible transformation when starting from *C*‐Tb_2_O_3_.^4^ Rare earth elements also form lower oxides like the monoxides *REO*, which can be obtained in the gas phase at high temperature under vacuum for all members of the series.1a EuO exists under standard conditions as well. Higher oxides in the compositional range *RE*O_*x*_ (1.5≤*x*≤2) are found for Ce, Pr, and Tb, due to their possible tetravalent oxidation state. Recently, even an oxidation state of +5 has been reported for praseodymium in PrO_4_ and the PrO_2_^+^ ion in a solid noble‐gas matrix and the gas phase, respectively.^5^ The dioxides *RE*O_2_ (*RE*=Ce, Pr, Tb) form under increased temperature and oxygen pressure, namely 573 K and around 1000 atm for TbO_2_, or through disproportionation from the oxides obtained by decomposition of precursors with O/*RE* ratios between 1.5 and 2. All known rare earth dioxides crystallize in the cubic fluorite structure (*Fm* $\bar{3}$ *m*) with, for example, *a=*522 pm for TbO_2_.^6^ Besides the mono‐, sesqui‐, and the dioxides, terbium also forms a homologous series of intermediate oxides, which are oxygen‐deficient and related to the fluorite structure. At high temperatures, wide composition ranges of a *σ*‐phase *RE*O_*x*_ (1.50<*x*<1.70) related to the *C*‐type structure, and an α‐phase *RE*O_*x*_ (1.72<*x*<2.00) related to the fluorite type structure exist. In the latter stoichiometric range, ordered intermediate phases with a narrow composition range Tb_2_O_2*n*−2 *m*_ (*n=*4…∞, *m=*1…8) exist at low temperatures.^1^, ^7^ *ι*‐Tb_7_O_12_ crystallizes in the trigonal (*R* $\bar{3}$ ), the *δ*‐phase Tb_11_O_20_ in the triclinic crystal system (*P* $\bar{1}$ ).^3^, ^8^ Both compounds are well characterized, forming a mixture, which represents the commercially available terbium oxide incorrectly known as “Tb_4_O_7_”. Other mixed‐valent terbium oxides are described with a more or less comprehensive characterization in literature.^3^, ^9^ However, no ordered intermediate within the composition range of the *σ*‐phase *RE*O_*x*_ (1.50<*x*<1.70) that is stable at room temperature has been reported up to now. To the best of our knowledge, no high‐pressure phase of a mixed‐valent rare earth oxide with a defined stoichiometry has been synthesized and characterized by means of its crystal structure, so far. For cerium oxide, a continuous range of solid‐solution intermediates, ranging between CeO_1.651_ and CeO_1.688_ (with Ce_3_O_5_=CeO_1.667_ in between), has been reported by Bevan to be present at around 1473 K in a gas mixture with defined partial pressures of CO_2_/O_2_, crystallizing in the body‐centered cubic *C*‐type structure.^10^ Furthermore, CeO_1.66_ has been found to crystallize in a cubic fluorite type superstructure (*Ia* $\bar{3}$ ) with *a=*11.111(2) Å.^11^

So far, the only reported evidence for the synthesis of a high‐pressure mixed‐valent binary terbium oxide was published by Lin‐Gun Liu in 1980, who described the formation of a high‐pressure phase with the composition TbO_1.75_ at pressures higher than 7 GPa and temperatures of 1073–1273 K. It was reported to crystallize in a cotunnite‐type structure, based on the data of a powder diffraction pattern; however, only half of the detected reflections, and therefore no distinct crystal structure, could be assigned.^12^ Whereas the composition of “Tb_3_O_5_” is sometimes used for mixtures of Tb_2_O_3_ and TbO_2_, no single phase of a terbium oxide with the composition Tb_3_O_5_ has been reported so far.

Although higher oxides of the rare earth metals can be obtained by various synthesis techniques, like flux growth methods or hydrothermal synthesis, the growth of single crystals of defined lanthanide oxides is still a difficult task.1a This is due to their high melting points (≈2773 K) and the insolubility of the lanthanide oxides in liquid solvents under ambient conditions. By the application of a high‐pressure/high‐temperature synthesis, utilizing a Walker‐type multianvil apparatus, we were able to establish a method for the synthesis of polycrystalline HT‐HP‐Tb_3_O_5_.

Here, we report in detail on the high‐pressure/high‐temperature synthesis and crystal structure of mixed‐valent HT‐HP‐Tb_3_O_5_ and its non‐centrosymmetric low‐temperature polymorph LT‐HP‐Tb_3_O_5_. The structural phase transition below 10 K was indicated by heat capacity measurements and confirmed by low‐temperature powder X‐ray diffraction. The structural changes at low temperature are described by a group‐subgroup relation. For HT‐HP‐Tb_3_O_5_, the temperature dependence of the magnetic susceptibility *χ* was measured at 100 Oe and 10 kOe. In order to confirm the presence of Tb^3+^ and Tb^4+^ in HP‐Tb_3_O_5_, the binding energies of the 4d electrons were investigated by X‐ray photoelectron spectroscopy.

## 2 Experimental Methods

**2.1 Synthesis**. HT‐HP‐Tb_3_O_5_ was synthesized under high‐pressure/high‐temperature conditions of 8 GPa and 1323 K in a Walker‐type multianvil apparatus. As starting materials, “Tb_4_O_7_” or an equimolar mixture of “Tb_4_O_7_” and Tb_2_O_3_ were found to be applicable. These were ground under an inert argon gas atmosphere and filled into boron nitride crucibles, which were then positioned inside of MgO octahedra and compressed by eight tungsten carbide cubes. HT‐HP‐Tb_3_O_5_ was obtained in form of air‐ and water‐resistant red‐brown crystals. Further experimental details on the synthesis are provided in the Supporting Information.

**2.2 Powder X‐ray Diffraction**. A powder XRD pattern of a flat polycrystalline sample of HT‐HP‐Tb_3_O_5_ was collected in transmission geometry using a Stoe Stadi P powder diffractometer (Stoe & Cie GmbH, Darmstadt, Germany) with a Mo source (Ge(111)‐monochromatized *K* ${}_{\alpha {}_{1}}$ radiation, *λ*=70.930 pm) operating at 50 kV and 40 mA. The powder diffraction pattern of Tb_3_O_5_ was recorded in the 2*θ* range 2.0–60.0° with a step size of 0.010°. Structural parameter refinement was carried out using the *INDEX & REFINE* tool within the Stoe WinX^POW^ software suite.^13^

**2.3 Single‐Crystal X‐Ray Diffraction**. Small single crystals of HT‐HP‐Tb_3_O_5_ were isolated by mechanical fragmentation and selected under an optical polarization microscope. Single‐crystal X‐ray diffraction data was collected at room temperature with a Bruker D8 Quest diffractometer (Photon 100) equipped with a microfocus source generator (Incoatec GmbH, Geesthacht, Germany), combined with multi layered optics (monochromatized Mo_*Kα*_ radiation, *λ*=71.073 pm). “Multi‐scan” absorption correction was applied with the program Bruker DIFABS‐2014/5.^14^ According to the systematic absences, the orthorhombic space group *Pnma* was derived. The structure was solved with SHELXS14b (version 2013/1) and refined (full‐matrix least‐squares against *F* ^2^) with SHELXL^15^ (version 2014/7) using Stoe X‐Step32^16^ (Revision 1.05b). The positional parameters of all atoms could be refined with anisotropic displacement parameters and were normalized utilizing the STRUCTURE TIDY routine in the PLATON software suite.^17^ A symmetry check using the ADDSYM routine within the PLATON software package suggested no changes. Final difference Fourier synthesis did not reveal any significant residual peaks. The relevant details of the data collection and evaluation are listed in Table S1 (Supporting Information). The positional parameters, isotropic and anisotropic equivalent displacement parameters, and interatomic distances are provided in Tables S3, S5, and S6. https://summary.ccdc.cam.ac.uk/structure-summary?doi=10.1002/chem201804006 1857704 contains the supplementary crystallographic data for HT‐HP‐Tb_3_O_5_. These data are provided free of charge by http://www.ccdc.cam.ac.uk/, CCDC/FIZ Karlsruhe deposition service.

**2.4 Elemental analysis**: EDX‐data (Energy‐dispersive X‐ray spectroscopy) was acquired for HT‐HP‐Tb_3_O_5_ using a scanning electron microscope (JEOL JSM6010‐LV) and a Bruker QUANTAX system equipped with a Peltier‐cooled Bruker XFlash 410‐M silicon drift detector. To avoid charging effects due to the insulating nature of the sample, the SEM was operated in low vacuum mode of 40 Pa. Spectra acquisition was performed at 15 kV accelerating voltage and 0.7 nA beam current, with an output count rate of 2100 cps. The lifetime during spectrum collection was 60 s and the deadtime was less than 3 %. The Bruker Esprit 1.9 software was used for spectra evaluation. Acquired spectra were evaluated standardless using the peak‐to‐background model and subsequent ZAF‐correction. Due to the standardless method, the totals are normalized to 100 %.

**2.5 Physical Properties**. Fragments of the button of HT‐HP‐Tb_3_O_5_ were attached to the sample holder rod of a Vibrating Sample Magnetometer unit (VSM) using Kapton® foil for measuring the magnetization *M*(T,H) in a Quantum Design Physical‐Property‐Measurement‐System (PPMS). The sample was investigated in the temperature range of 2.5–300 K with magnetic flux densities up to 80 kOe. For the heat capacity measurements, one piece of the sample (5.877 mg) was fixed to a pre‐calibrated heat capacity puck using Apiezon N grease and measured in the temperature range of 2.1 to 300 K.

**2.6 Low‐Temperature Powder X‐Ray Diffraction**. Low‐temperature powder diffraction data was collected at the Swiss‐Norwegian Beam Lines (BM01) at the ESRF (Grenoble, France) using a Dectris Pilatus2M detector at 2(1) and 300(1) K. The wavelength was set to 0.6967 Å. Low temperature was achieved and controlled with an in‐house developed He blower. Experimental powder diffraction data was integrated and processed using the PyFAI module;^18^ structural parameters were refined with the FullProf software.^19^

**2.7 X‐Ray Photoelectron Spectroscopy (XPS)**. X‐ray photoelectron spectra were measured using an Axis‐Ultra spectrometer (Kratos, Manchester, UK) in ultrahigh vacuum (pressure <10^−7^ Pa). Monochromatic Al_*Kα*_ radiation (*hυ*=1486.6 eV) was used with a 12 kV accelerating voltage and 10 mA emission current. The charge neutralizer was run with a filament current of 1.8 A, a charge balance of 2.25 V, and a filament bias of 1.0 V. A pass‐energy filter of 40 eV was applied to the hemispherical analyzer (electrostatic lens mode), enabling high‐energy resolution for narrow scans with a step size of 0.1 eV at a dwell time of 4 s. Commercially available Tb_6_O_11_ (pressed into a pellet) and pieces of HT‐HP‐Tb_3_O_5_ were attached to the XPS sample holder using conductive carbon tape. An area of approximately 1×2 mm on the sample surface was irradiated at an X‐ray emission angle of 30° relative to the sample. Due to different static charge phenomena of the samples, the C 1s signal, caused by organic surface contaminations, was used as an internal reference and its binding energy was assumed to be 285.0 eV (referenced to the Fermi level) for all samples. The positional parameters for HT‐HP‐Tb_3_O_4_ and LT‐HP‐Tb_3_O_5_ based on synchrotron powder X‐ray diffraction data are provided in Table S4.

## 3 Results and Discussion

### 3.1 Room‐temperature single‐crystal X‐ray diffraction.

The crystal structure of HT‐HP‐Tb_3_O_5_ was solved from single‐crystal X‐ray diffraction data using Direct Methods. Free refinement of the atomic occupancy factors resulted only in small deviations from full occupancy (<0.2 %). The composition of HT‐HP‐Tb_3_O_5_ was confirmed by EDX measurements. Although the measurements had to be performed in low vacuum due to the insulating character of the sample crystal of HT‐HP‐Tb_3_O_5_, the mean composition obtained by microprobe analysis equaled to Tb_1.00(3)_O_1.7(2)_.

**Figure 1:**
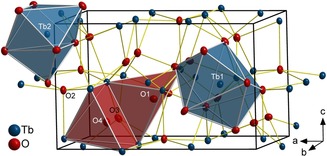
Ball‐and‐stick model representing the crystal structure of HT‐HP‐Tb_3_O_5_ (orthorhombic, space group *Pnma*) drawn with 90 % displacement ellipsoids viewed close to [0 $\bar{1}$ 0]. Coordination polyhedra around the terbium cations are depicted in blue shades and around the oxygen atoms O1 and O4 (the latter exemplarily for all square pyramidally coordinated oxygen atoms O2, O3, and O4) in red.

HT‐HP‐Tb_3_O_5_ crystallizes in the orthorhombic centrosymmetric space group *Pnma* with the cell parameters *a=*9.7930(6), *b=*6.7064(4), and *c=*6.0423(3) Å. The anisotropic displacement parameters show a slight but not distinct ellipsoidal expansion for the terbium cations, and an almost isotropic displacement for the oxygen ions, except for O2, which shows the strongest ellipsoidal expansion (see Table S5 in Supporting Information). A representation of the crystal structure with 90 % displacement ellipsoids is given in Figure 1. HT‐HP‐Tb_3_O_5_ is isopointal with U_3_S_5_,^20^ the structural anti‐type of β‐Yb_5_Sb_3_.^21^ The comparison of structural features is equally valid for both structure types. The crystal structure of HT‐HP‐Tb_3_O_5_ is also closely related to some oxometallates based on the structure type of (Sr_0.5_La_0.5_)_2_BeLaO_5_, incorporating beryllium atoms in tetrahedral interstices formed by oxygen anions.^22^

More than 280 entries for the space group type *Pnma*, Pearson symbol *oP*32, and Wyckoff sequence *d* ^2^ *c* ^4^ are listed in the Pearson data base, and they have been labeled with the structure types β‐Yb_5_Sb_3_, Y_5_Bi_3_, Y_2_HfS_5_, and K_2_PrCl_5_. Keeping the distinct differences in chemical bonding in mind, these compounds should be called isopointal rather than isotypic. Around fifty compounds were reported to crystallize in the β‐Yb_5_Sb_3_‐type structure. More than half of them are binary compounds, however some ternary ordered substitution variants of the β‐Yb_5_Sb_3_‐type structure are reported in literature as well, for example, Y_2_HfS_5_.^23^ Most of these compounds are intermetallic phases, namely antimonides, bismuthides, and tellurides, but also arsenides, selenides, and sulfides, for example, U_3_S_5_ 20 and U_3_Se_5_.^24^ However, no element of the second period of the periodic table has been found to be included in a crystalline compound exhibiting a β‐Yb_5_Sb_3_‐type structure.^25^ Of this large family of crystal chemically different orthorhombic phases, especially the sulfide Y_2_HfS_5_ underlines the charge ordering in HT‐HP‐Tb_3_O_5_: Tb^3+^Tb^3+^Tb^4+^(5O^2−^) and Y^3+^Y^3+^Hf^4+^(5S^2−^).

By the application of high‐pressure/high‐temperature conditions, we were able to synthesize a new terbium oxide, HT‐HP‐Tb_3_O_5_, crystallizing in the structure of its higher homologues, for example, U_3_S_5_, U_3_Se_5_, and Y_2_HfS_5_.^23^ In an extended view, HT‐HP‐Tb_3_O_5_ thereby follows the pressure‐homologue rule for stable phases in ionic solids, according to the principle that high‐pressure conditions favor the formation of phases with a structure type of their heavier homologues.^26^

Many compounds formerly ascribed to crystallize in the β‐Yb_5_Sb_3_‐type structure were found to comprise hydrogen atoms, for example, in *A*_5_ *Pn*_3_H (*A*=Ca, Sr, Eu, Yb; *Pn*=Sb, Bi), thereby forming hydrides in a stuffed version of the β‐Yb_5_Sb_3_ structure or as well in the Mn_5_Si_3_ structure. Therefore, these compounds are to be assigned with the Ca_5_Sb_3_F_*x*_‐type structure.^27^ As many compounds are known to inherit additional atoms (H, F, Cl, Br) in their tetrahedral interstices,^28^ special attention has been paid on the residual electron density in the refined structure of HT‐HP‐Tb_3_O_5_. However, no significant residual electron density could be found within the tetrahedral sites formed by the oxygen atoms in the structure of HT‐HP‐Tb_3_O_5_.

**Figure 2:**
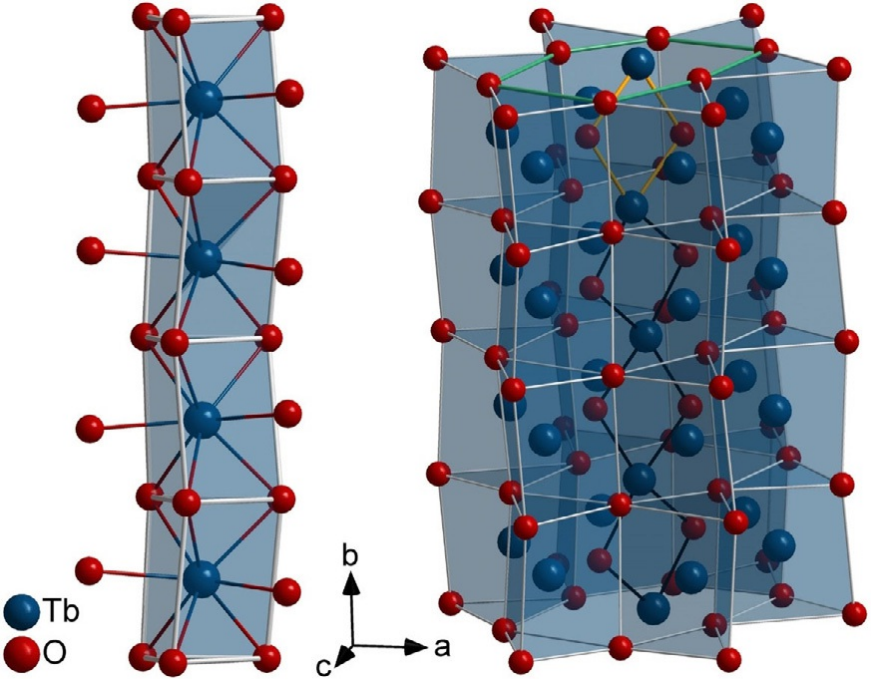
Chain(s) formed by trigonal prisms in the crystal structure of HT‐HP‐Tb_3_O_5_; Left: Infinite chain of trigonal prisms formed by oxygen atoms around Tb1 running along the *b*‐axis, showing the buckling of these chains. Right: Six chains of trigonal prisms forming a distorted hexagonal channel (one hexagonal base plane is highlighted in green on top). Within the channel, two terbium and two oxygen atoms form a rhomb on each plane (one highlighted in orange on top), being interconnected via the terbium atoms to form a zig‐zag chain along the hexagonal channel.

The crystal structure of HT‐HP‐Tb_3_O_5_ consists of trigonal prisms, formed by the oxygen atoms, with a terbium atom approximately in the center of these prisms. The prisms share triangular faces to form interconnected infinite columns parallel to [0 1 0] (Figure 2 left). The trigonal prisms further share edges with neighboring prism chains to form a three‐dimensional network, with large and strongly deformed hexagonal channels (Figure 2 right). Each thereby‐formed hexagonal prism contains two oxygen atoms O1 in the central plane and one terbium atom Tb1 offset from the center of each hexagonal base plane. The alignment of these displacements is reversed for every second hexagonal base plane, forming zig‐zag chains. The atoms within the hexagonal channel form rhombs by two oxygen and two terbium atoms each, the latter being shared with the next rhombs to produce an infinite chain parallel to the crystallographic *b*‐axis.

**Figure 3:**
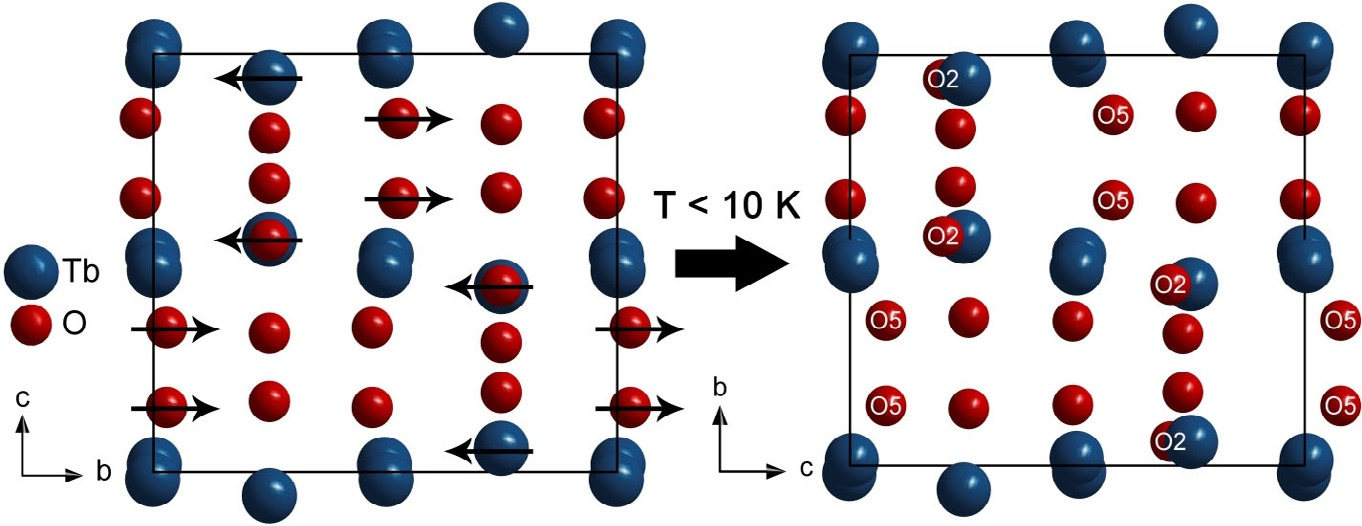
Layers of atoms perpendicular to [0 1 0], viewed along [1 0 0] in the crystal structures of HP‐Tb_3_O_5_. Left: HT‐HP‐Tb_3_O_5_; The layers around *y=*0 and *y=*0.5 show a buckling characteristic for the β‐Yb_5_Sb_3_‐type structure. The atom displacements in the transition to the low‐temperature polymorph are indicated by arrows. Right: LT‐HP‐Tb_3_O_5_; The oxygen atoms O5 (equaling O1 in HT‐HP‐Tb_3_O_5_) and O2 are shifted along [0 0 1] (equaling [0 1 0] in HT‐HP‐Tb_3_O_5_).

Alternatively, the structure of HT‐HP‐Tb_3_O_5_ can be described in terms of layers of atoms perpendicular to [0 10 ] (see Figure 3 left). The layers at *y=*1/4 and *y=*3/4 are planar (mirror planes) and contain the oxygen atoms forming the trigonal prisms and the hexagonal channels, as well as the terbium atoms off center of these elongated hexagons (see Figure S3). The other atoms form buckled layers between *y*=±0.0283(3) and 0.4717(3)≤*y*≤0.5283(3). They contain the terbium atoms forming hexagons with two oxygen atoms within, symmetrically disposed from the center of the hexagons. The nature of the layer buckling is characteristic for the β‐Yb_5_Sb_3_‐type structure and allows for a clear distinction from the isopointal compound Y_5_Bi_3_.^29^ Both structures are superstructures of the Rh_5_Ge_3_‐type (*klassengleiche* transition of index 2 from *Pbam* to *Pnma* through a doubling of the unit cell).^30^ The structure of HT‐HP‐Tb_3_O_5_ can further be distinguished from the intermetallic compounds like Rh_5_Ge_3_ or Tm_3_Ga_5_ by comparing the mean axial ratios (HT‐HP‐Tb_3_O_5_: *a*/*b=*1.46, *c*/*b=*0.90, *a*/*c=*1.61; Tm_3_Ga_5_: *a*/*b=*1.18, *c*/*b=*0.63, *a*/*c=*1.88; U_3_S_5_: *a*/*b=*1.45, *c*/*b=*0.91, *a*/*c=*1.58).^31^ An excellent overview on the many coloring and deformation variants of these orthorhombic structures is given by Gladyshevskii et al.^32^

In the crystal structure of HT‐HP‐Tb_3_O_5_, Tb2 is irregularly coordinated by seven oxygen atoms with distances of 215 to 234 pm. The coordination symmetry can be described as a strongly distorted mono‐capped octahedron (see Figure 1 and Figure S2 top right). Tb1 is coordinated by eight oxygen atoms with interatomic distances between 233 and 252 pm in form of a bi‐capped trigonal prism (Figures 1 and S2 top left). These prisms form the aforementioned infinite chains along [0 1 0] through face sharing. The oxygen anions are coordinated by four (O1) and five (O2, O3, O4) terbium cations in form of distorted tetrahedra and square‐pyramids, respectively (Figure 1 and Figure S2 bottom). Calculations of the effective coordination numbers (ECoN) according to Hoppe^33^ clearly confirm the respective coordination numbers of the ions in HT‐HP‐Tb_3_O_5_.

For comparison, the coordination of the rare earth ions in the constitutionally nearest normal‐pressure terbium oxide *ι*‐Tb_7_O_12_ is lower, with six for Tb1 and seven for Tb2. The coordination of the rare earth ions in the *A*‐, *B*‐, and *C*‐form of the sesquioxides is similarly low with six (*C*‐type), six and seven (*B*‐type), and seven (*A*‐type). Hence the pressure‐coordination rule is fulfilled, stating that the coordination number, especially of the cations, is higher for high‐pressure polymorphs in comparison to their low‐pressure analogues.^26^

Though the crystal structure of HT‐HP‐Tb_3_O_5_ can hardly be described as an oxygen deficient defect variant of the fluorite structure, similar features as in the anion‐deficient, fluorite‐related structures are found. Several tetrahedral, as well as square pyramidal coordination spheres formed by the terbium cations are centered by an oxygen vacancy.

Further facts on the structural details of HT‐HP‐Tb_3_O_5_ can be found in the Supporting Information.

### 3.2 Physical properties

**Figure 4:**
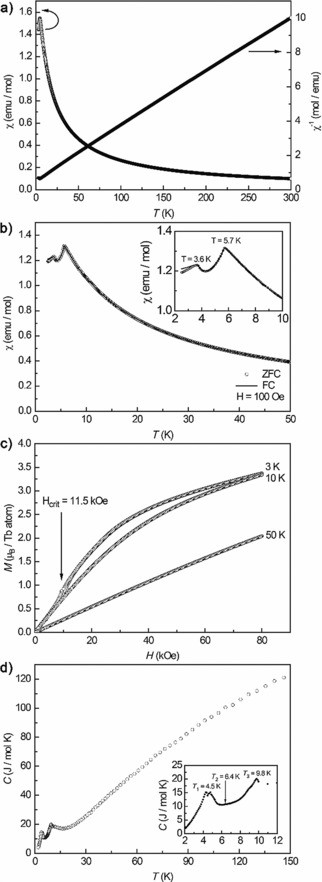
Physical properties of HP‐Tb_3_O_5_: a) Temperature dependence of the magnetic susceptibility (*χ* and *χ* ^−1^ data) measured at 10 kOe. b) Susceptibility measurements in zero‐field‐ and field‐cooled mode measured at 100 Oe; the inset shows a zoom of the low temperature region. c) Magnetization isotherms at 3, 10, and 50 K. d) Temperature dependence of the specific heat; the inset showing a zoom of the low temperature region.

For HP‐Tb_3_O_5_, the temperature dependence of the magnetic susceptibility *χ* was measured at 10 kOe (Figure 4 a). A Curie–Weiss fit of the reciprocal magnetic susceptibilities in the paramagnetic region (50–300 K) was used to determine the effective magnetic movement *μ*_eff_ and the Weiss constant. An effective magnetic moment of *μ*_eff_=9.21(2) *μ*_B_ per formula unit was determined, which is well below the theoretical magnetic moment *μ*_calc_=9.72 *μ*_B_ for a Tb^3+^ cation. It is, however, in line with the expectations, since, according to the sum formula, HP‐Tb_3_O_5_ contains two Tb^3+^ and one Tb^4+^ cation. Therefore, the effective magnetic moment has to be calculated using the squared moments with the respective fractions according to Equation (1):

(1) $$\mu {}_{\mathrm{calc}}=\sqrt{\frac{\left(2\;\times \;{\left(9.72\;\mu_{B}\right)}^{2}+{1\;\times \left(7.94\;\mu_{B}\right)}^{2}\right)}{3}}=9.17\;\mu {}_{B}$$

For Tb^4+^, the calculated magnetic moment of the isoelectronic Gd^3+^ (*μ*_calc_=7.94 *μ*_B_) must be used, leading to the calculated moment of *μ*_calc_=9.17 μ_B_ for Tb_3_O_5_. The calculated magnetic moment matches the observed effective magnetic moment very well; the differences can be explained by the presence of small amounts of a Tb_6_O_11_ impurity, as observed in powder X‐ray diffraction experiments. The calculated Weiss constant is negative, indicating dominant anti‐ferromagnetic interactions in the paramagnetic region.

Additionally, low‐field measurements with an external field of 100 Oe were performed in a zero‐field‐cooled/ field‐cooled mode (ZFC/FC) and are shown in Figure 4 b. Here, two antiferromagnetic transitions arise at Néel temperatures of *T*_N,1_=3.6(1) K and *T*_N,2_=5.7(1) K (inset of Figure 4 b). The anomaly at the higher temperature is caused by the presence of Tb_6_O_11_ (=TbO_1.833_) as trace impurity, which has an ordering temperature of *T* ≈6 K according to the literature.^34^ Therefore, the transition at *T*_N,1_=3.6(1) K is the intrinsic ordering temperature for HP‐Tb_3_O_5_. To prove this, the sample was further studied by heat capacity measurement in zero magnetic field (Figure 4 d). The *C*_P_ data reveals that the first anomaly at low temperatures at *T*_1_=4.1(1) K is strong compared to the feature visible at *T*_2_=6.4(1) K, proving that the magnetic low‐temperature transition originates from the majority component of the sample, HP‐Tb_3_O_5_. In addition, another lambda‐shaped peak is visible at *T*_3_=9.8 K, which could not be detected in the susceptibility measurements. Due to the strong nature of this feature, an intrinsic structural phase transition of HP‐Tb_3_O_5_ seems possible (see below).

Furthermore, magnetization isotherms below and above the ordering temperature (3, 10, and 50 K) were measured in order to investigate the stability of the AFM ground state, and to determine the saturation magnetization at 3 K and 80 kOe (Figure 4 c). The magnetization isotherm recorded at 3 K shows a pronounced spin‐reorientation (also known as metamagnetic step) at a critical field of *H*_crit_=11.5(1) kOe. At this field, the AFM ground state switches to a ferromagnetic (FM) ground state with a small hysteresis upon field reduction. The magnetization isotherm at 10 K shows a pronounced curvature, due to the temperature being close to the transition temperature, whereas the isotherm recorded at 50 K shows a linear field dependency, as expected for a paramagnetic material. The saturation magnetization at 3 K and 80 kOe was determined to be *μ*_sat_=10.1(1) *μ*_B_ being lower than the expected moment of *μ*_sat,calc_=25 *μ*_B_ according to *g*_J_ × *J*.

### 3.3 Low‐temperature powder X‐ray diffraction

**Figure 5:**
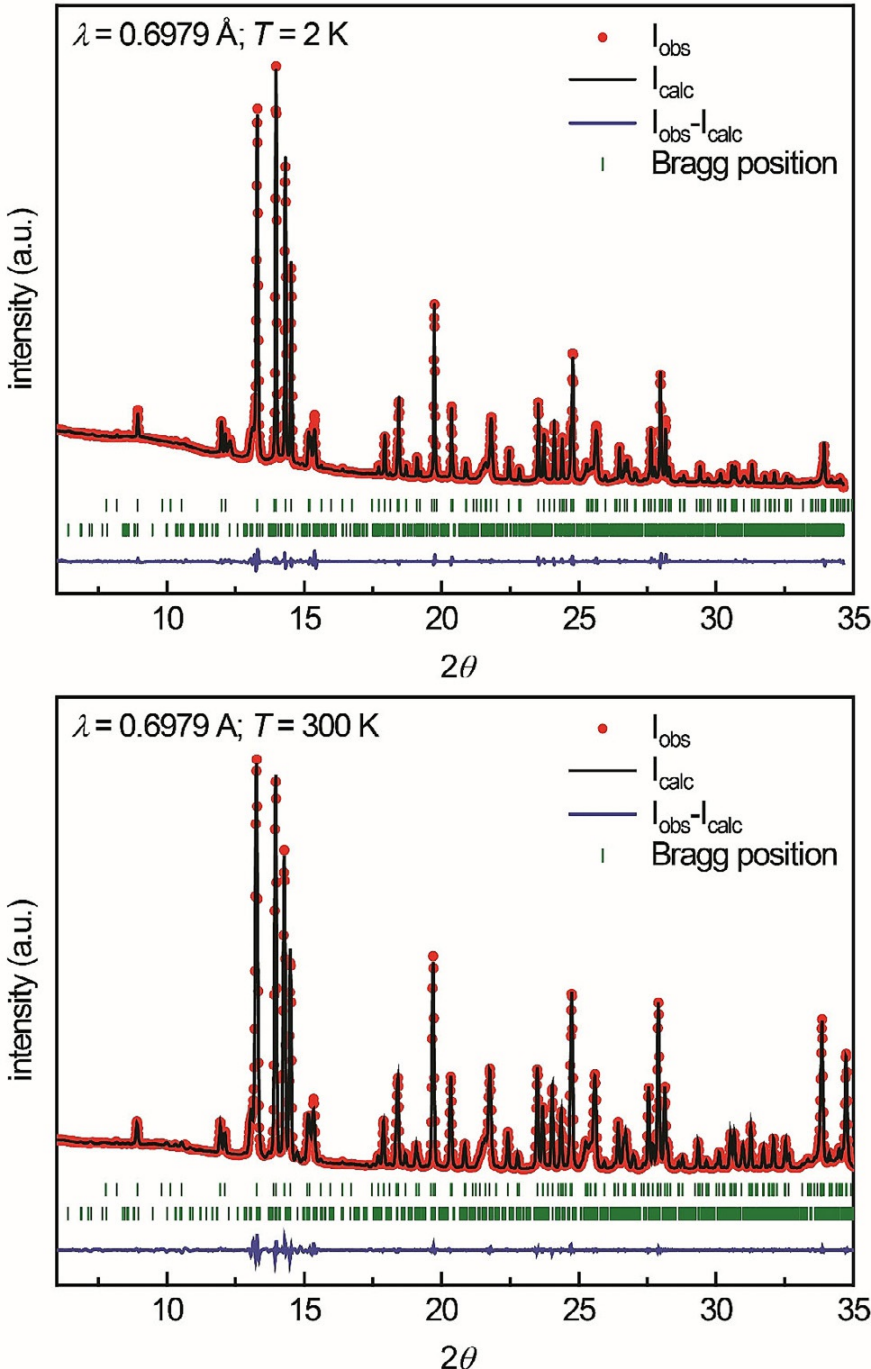
Synchrotron powder X‐ray diffraction data of HP‐Tb_3_O_5_. Top: Data acquired at 2 K, the upper set of Bragg positions corresponds to LT‐HP‐Tb_3_O_5_ (*Pna*2_1_), the lower set to Tb_6_O_11_ (*P* $\bar{1}$ ). Bottom: Data acquired at 300 K, the upper set of Bragg positions corresponds to HT‐HP‐Tb_3_O_5_ (*Pnma*), the lower set to Tb_6_O_11_ (*P* $\bar{1}$ ).

Since a structural phase transition below 10 K–with respect to the heat capacity measurements–seemed possible, synchrotron powder diffraction data was collected at 2(1) and 300(1) K. The 300 K powder pattern could be fitted with HT‐HP‐Tb_3_O_5_ as the main phase and traces of Tb_6_O_11_, as already evident from the susceptibility measurements (see above). The crystallographic parameters of HT‐HP‐Tb_3_O_5_, obtained by single‐crystal X‐ray diffraction, were used for the Rietveld refinement. Due to the structural complexity of Tb_6_O_11_ (*P* $\bar{1}$ , 24 Tb sites, 44 O sites),8c the impurity phase was only implemented as an additional full pattern fit. Figure 5 (bottom) depicts the refinement of the 300 K diffraction pattern; the obtained crystallographic parameters are in line with the values from the single‐crystal determination and are listed in Table S2 (Supporting Information). These parameters were initially used to refine the low‐temperature data, recorded at 2 K. It quickly became evident from the difference line (*I*_obs_−*I*_calc_) that the fit with the *Pnma* structure is not correct at low temperatures, and a structural change (see heat capacity measurements) has to take place. However, no superstructure reflections are visible, ruling out *klassengleiche* transitions. Hence, only a *translationengleiche* transition retaining the orthorhombic crystal system is possible. The most reasonable symmetry reduction is the loss of the mirror plane in *Pnma*, leading to space group *Pn*2_1_ *a* (non‐standard setting of *Pna*2_1_). The structure of LT‐HP‐Tb_3_O_5_ was therefore refined with this acentric space group leading to significantly enhanced results. The fit is depicted in Figure 5 (top), the obtained crystallographic parameters are listed in Table S2; details of the group‐subgroup relation are given below.

**Figure 6:**
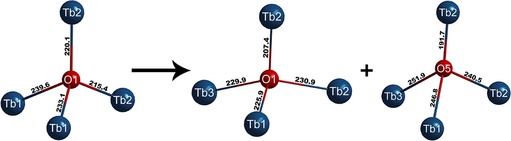
Coordination of the oxygen atoms O1 and O5 and interatomic distances (pm) in the crystal structures of HT‐HP‐Tb_3_O_5_ (left) and LT‐HP‐Tb_3_O_5_ (middle and right). Due to the loss of the mirror plane, the oxygen atom O1 in HT‐HP‐Tb_3_O_5_ splits into two crystallographic positions O1 and O5, accompanied by strong distortions of the tetrahedral coordination spheres.

The crystal structure of the low‐temperature polymorph LT‐HP‐Tb_3_O_5_ only shows some slight differences compared to the high‐temperature polymorph HT‐HP‐Tb_3_O_5_. Minor changes of the lattice parameters result in a slightly decreased unit cell volume of 0.3946 nm^3^ (−0.56 % compared to 0.3968 nm^3^ in HT‐HP‐Tb_3_O_5_). The atomic coordinates of the rare earth cations and the oxygen anions O3 and O4 nearly stay the same. However, the oxygen ions O1, but especially O2 as well as O5 (O5 equaling O1 in HT‐HP‐Tb_3_O_5_) are shifted along the crystallographic *c*‐axis. This shift causes the loss of the mirror plane and leads to a change in the nature of the buckling of the atom layers perpendicular to [0 0 1], as is shown in Figure 3. The layers around *z=*1/4 and *z=*3/4 are no longer planar. While the coordination of the rare earth cations and the oxygen anions O1‐O4 in the low‐temperature polymorph equals the coordination in HT‐HP‐Tb_3_O_5_, the oxygen ions O5 in LT‐HP‐Tb_3_O_5_ exhibit a 1+3 coordination, rather than the fourfold coordination of the corresponding O1 in the crystal structure of HT‐HP‐Tb_3_O_5_. This results in a major distortion of the tetrahedral‐like coordination sphere into an almost trigonal‐planar coordination, as depicted in Figure 6. Similarly, all coordination polyhedra in the crystal structure of the low‐temperature polymorph are slightly more distorted than in the high‐temperature polymorph HT‐HP‐Tb_3_O_5_. The trigonal prism chains, as well as the hexagonal prism chains along [0 0 1], are hardly affected by the structural phase transition; however, the chains of rhombs inside the hexagonal channels are distorted as they are built up by the oxygen atoms O1 and O5.

For the low‐temperature phase, we can draw back to charge ordering, which has been observed in a variety of mixed alkali metal (*A*) rare earth (*RE*) halides (*X*) of compositions *A*_2_ *REX*_5_ (fluorides, chlorides, bromides, and iodides). The early investigated prototypes are K_2_SmF_5_ 35 and K_2_PrCl_5_.^36^ Although the existence ranges for these halides are not entirely known, it is obvious that the smaller fluorides adopt the non‐centrosymmetric *Pn*2_1_ *a* phase, whereas the larger halides crystallize with the centrosymmetric *Pnma* variant. Thus, the smaller fluoride anions create some kind of an internal (chemical) pressure, leading to the loss of the mirror plane and enabling better packing density. One should note that, in the Pearson data base,^37^ the isopointal *Pnma* phases are labeled with the prototypes Y_2_HfS_5_ and K_2_PrCl_5_.

### 3.4 Group‐subgroup relation

**Figure 7:**
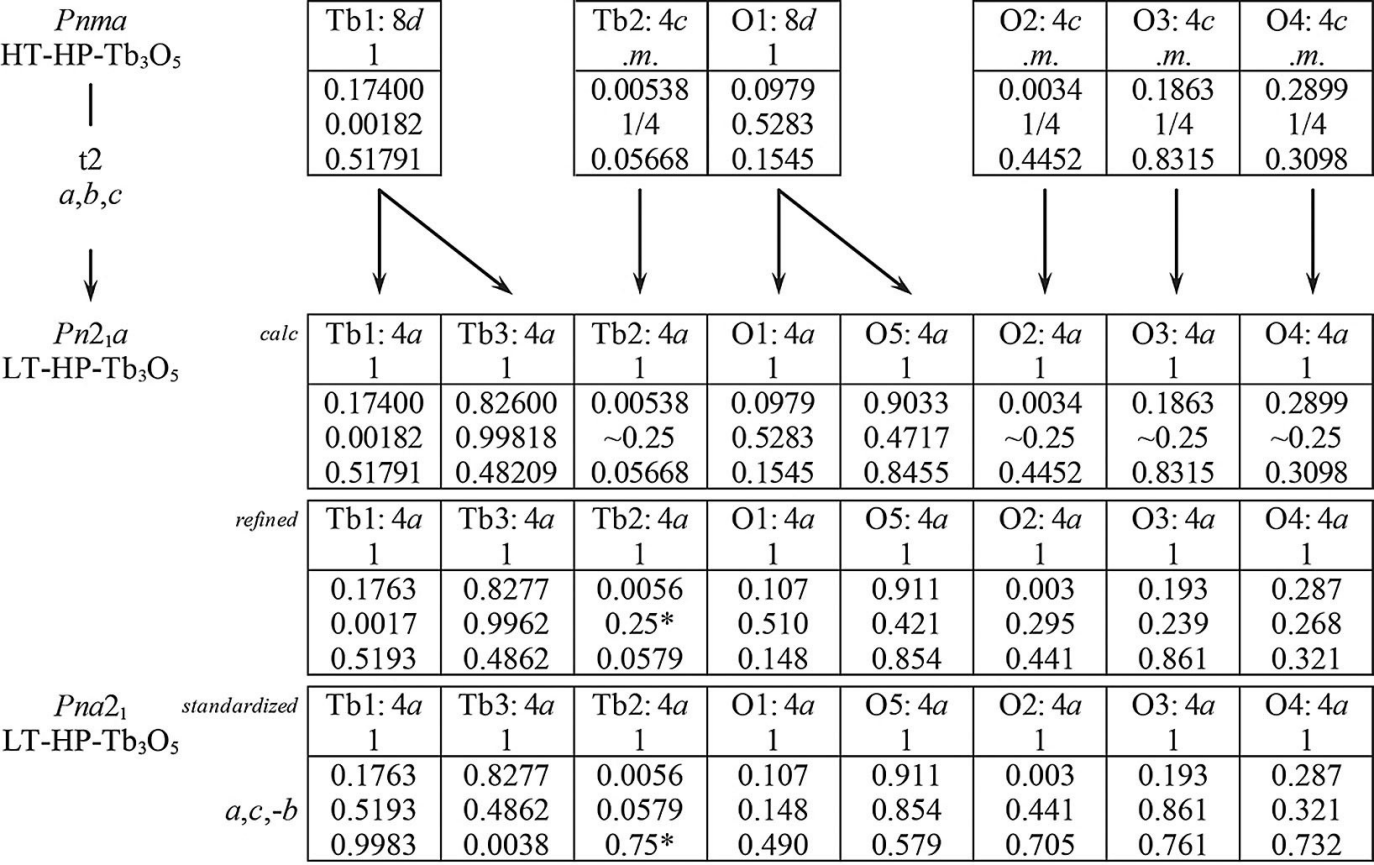
Group‐subgroup scheme in the Bärnighausen formalism^39^ for the structures of HT‐HP‐Tb_3_O_5_ and LT‐HP‐Tb_3_O_5_. The indices for the *translationengleiche* (t) symmetry reductions, the unit cell transformations, the evolution of the atomic parameters, and the standardized atomic parameters are given.

The group‐subgroup relation of HT‐HP‐Tb_3_O_5_ and LT‐HP‐Tb_3_O_5_ is shown in Figure 7. As described before, no superstructure reflections are visible in the 2 K measurement, therefore only a *translationengleiche* transition is possible. In HT‐HP‐Tb_3_O_5_, the Tb2, O2, O3, and O4 atoms are located on a mirror plane with the fixed atomic parameter *y*=1/4. Removing this mirror plane leads to space group *Pn*2_1_ *a* (non‐standard setting of *Pna*2_1_), which allows for additional degrees of freedom for all atoms mentioned before. Whereas the Tb atoms exhibit only minor shifts off of their calculated atomic positions, rather strong shifts are observed for O2, O3, and O4 in LT‐HP‐Tb_3_O_5_ (Table S4 in Supporting Information). The Tb1 and O1 atoms found on the 8*d* site in HT‐HP‐Tb_3_O_5_ split into two fourfold positions, also leading to additional degrees of freedom with respect to structural distortions. The obtained interatomic distances for both phases are listed in Table S6. Calculations of the bond valence sums of HT‐HP‐Tb_3_O_5_ and LT‐HP‐Tb_3_O_5_ indicate no temperature‐dependent valence phase transition. In contrast to other compounds that contain ions exhibiting more than one possible oxidation state, like in Eu_2_Pt_6_Al_15_,^38^ HP‐Tb_3_O_5_ shows no significant changes in the valences of the respective ions according to calculations of the bond valence sums (see below).

### 3.5 X‐ray photoelectron spectroscopy (XPS)

**Figure 8:**
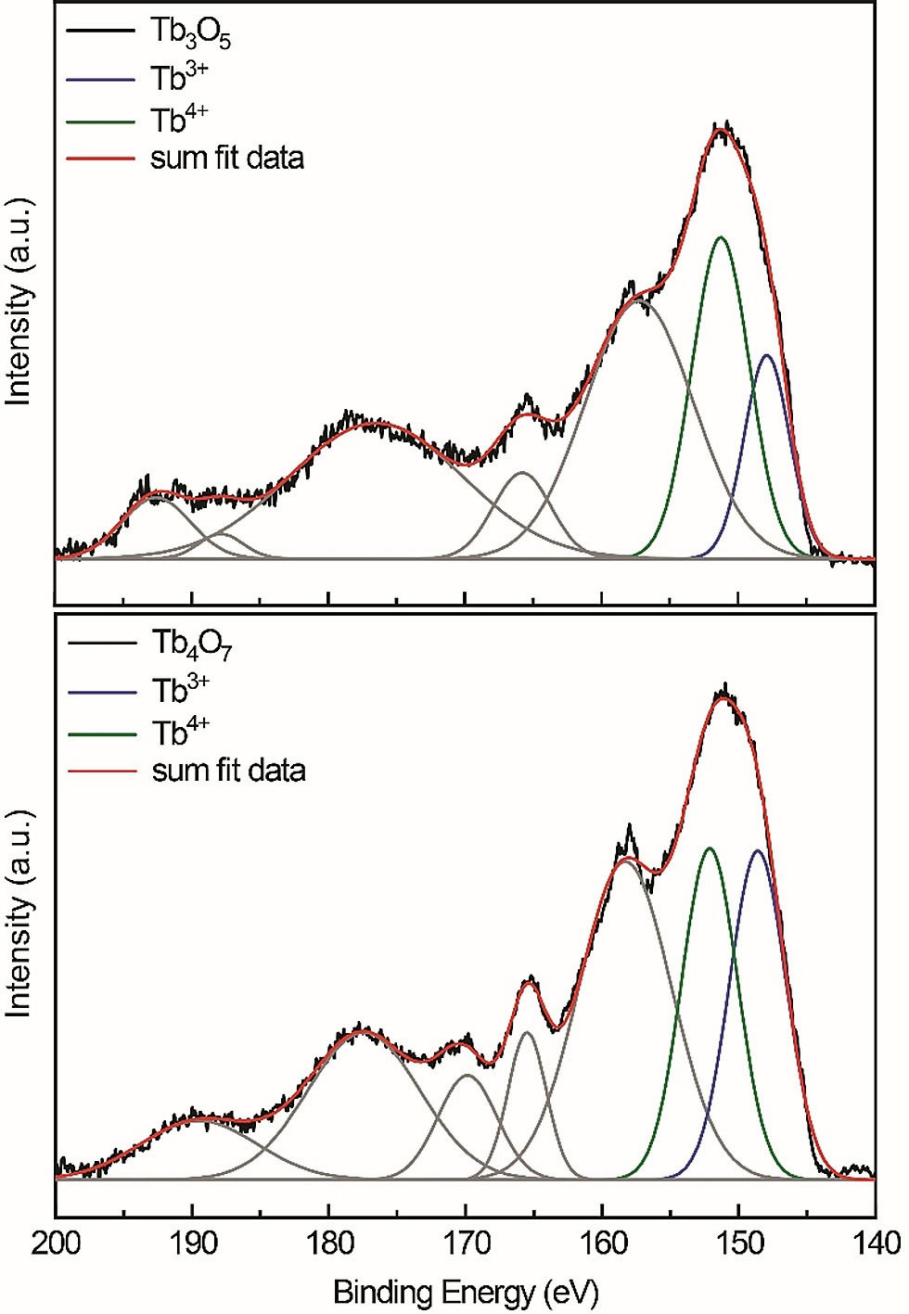
X‐ray photoelectron spectroscopic (XPS) data (black lines) of HT‐HP‐Tb_3_O_5_ (top) and Tb_4_O_7_ (bottom). The signals caused by the Tb^3+^ cations are shown in blue, the ones of the Tb^4+^ cations in green. Charge‐transfer satellites are depicted in grey. The sum of all signals is shown in red.

The binding energies of the 4d electrons of HT‐HP‐Tb_3_O_5_ and Tb_4_O_7_ were investigated in order to confirm the presence of Tb^3+^ and Tb^4+^. The 4d binding energies for Tb^3+^ in Tb_4_O_7_ are found at around ≈149 eV,^40^ and the ones for Tb^4+^ at around ≈156 eV.^40^ Additionally, the main peaks are accompanied by charge‐transfer satellites with ≈4 eV higher energies. These can be attributed to O 2p→Tb 4f excitations.40a The obtained and fitted spectra of HT‐HP‐Tb_3_O_5_ and Tb_4_O_7_ are depicted in Figure 8, the extracted binding energies are listed in Table 1. Both spectra are comprised of several peaks, which were fitted using Gaussian profiles and the XPS analytical tool implemented in the Origin program package. The relative ratios of the Tb^3+^/Tb^4+^ signals were fixed according to the sum formulas during the fit; the peak positions and FWHM, however, were freely refined. In both compounds, the signal at the lowest binding energy was attributed to originate from the Tb^3+^, the following by the Tb^4+^ cations. In comparison with the literature, all signals are shifted to slightly lower binding energies. Since the spectra were calibrated towards the C 1s lines, they can be compared to each other; however, it is not assured that the calibration for the literature compounds was done with the same carbon species.

**Table 1:** Binding energies (eV) of the 4*d* electrons determined by XPS of HT‐HP‐Tb_3_O_5_, Tb_4_O_7_ and data from the literature. The determined error is ±0.2 eV.

Compound	4d(Tb^3+^)	4d(Tb^4+^)	Satellite charge‐transfer transitions				
HT‐HP‐Tb_3_O_5_	147.8	151.2	157.2	165.7	176.6	–	This Work
Tb_4_O_7_	148.6	152.1	158.3	165.6	177.6	–	This Work
Tb_2_O_3_	148.4	–	–	–	173.7	181.5	40a
Tb_4_O_7_	148.9	156.5	–	164.2	177.0	188.5	40a
TbO_2_	–	157.3	–	164.2	177.0	188.4	40a

### 3.6 Charge distribution calculations

Calculations of the bond valences of HT‐HP‐Tb_3_O_5_ and LT‐HP‐Tb_3_O_5_ were performed according to the *BLBS* 41 (bond‐length/bond‐strength, parameters listed in Ref. 42, obtained from Ref. 43 for Tb^4+^ on Tb2 sites and Ref. 41c for Tb^3+^ on Tb1 and Tb3 sites) and the *CHARDI* 33, 41c,41d, 44 (charge distribution in solids) concept. The results are listed in Table 2. Within the limits of these concepts, the calculated charges correlate well with the expected values, although the values obtained for the Tb cations differ strongly depending on the calculation method used. Whereas the results based on the *CHARDI* calculations propose approximately equal charges for the two/three crystallographically independent Tb cations, the values obtained with the *BLBS*‐based calculations state a much higher positive charge of the Tb2 ion compared to the Tb1 (and Tb3) ions. This can be related to the fact that four Tb1 atoms coordinate the oxygen vacancy site in the crystal structure of HT‐HP‐Tb_3_O_5_, whereas this vacancy is coordinated by only one Tb2 atom. This corresponds to the results found for Ce_7_O_12_, where the charge of the two electrons left by the oxygen atom when forming an O vacancy delocalizes on the surrounding Ce sites. Therefore, the three‐vacancy‐coordinating Ce1 sites are affected to a higher extent than the only one‐coordinating Ce2 atom.^45^ Investigations of the electronic structure of the isostructural compounds U_3_S_5_ and U_3_Se_5_ based on the ionic radii and XPS measurements showed the same mixed valency for the uranium ions. The formula of these compounds can therefore be written as U^3+^U^3+^U^4+^(S^2−^)_5_ and U^3+^U^3+^U^4+^(Se^2−^)_5_, respectively.8a, 9b,9c, 20a Furthermore, a comparison to ordered substitution variants of the U_3_S_5_ structure type like Y_2_HfS_5_ 23 is very interesting. The formal charges of the cations in the isostructural compound Y_2_HfS_5_ can be clearly stated to be +3 for Y and +4 for Hf, with the yttrium cations located on the Tb1 sites and the hafnium cations on the Tb2 sites. This corresponds well to the results obtained for the valences of the terbium ions in HT‐HP‐Tb_3_O_5_ from *BLBS* calculations. Taking all of the mentioned aspects into account, together with the results of the *CHARDI* calculations and the fact that the HT‐HP‐Tb_3_O_5_ sample shows a strong optical absorption in the visible spectrum, we conclude that HT‐HP‐Tb_3_O_5_ exhibits a Class 2^46^ inhomogeneous mixed valence of the terbium cations.

**Table 2:** Charge distribution in HT‐HP‐Tb_3_O_5_ and LT‐HP‐Tb_3_O_5_ calculated according to the bond‐length/bond‐strength (Σ*V*) ^[41]^ and the *CHARDI* (Σ*Q*) ^[33, 41c,d, 44]^ concept.

		Tb1	Tb2	Tb3	O1	O2	O3	O4	O5
HT‐HP‐Tb_3_O_5_	Σ*V*	+2.96	+4.23		−2.20	−1.92	−1.81	−2.02	
	Σ*Q*	+3.34	+3.32		−2.02	−2.00	−1.85	−2.09	
LT‐HP‐Tb_3_O_5_	Σ*V*	+3.05	+4.65	+3.21	−2.43	−2.14	−1.97	−2.12	−2.25
	Σ*Q*	+3.32	+3.36	+3.32	−2.20	−2.00	−1.88	−2.07	−1.85

## 4 Conclusion

With HP‐Tb_3_O_5_, a new binary rare earth oxide could be synthesized and characterized in detail. The access to its low‐temperature polymorph LT‐HP‐Tb_3_O_5_ is exclusively possible through the displacive phase transition upon cooling the high‐temperature‐high‐pressure modification HT‐HP‐Tb_3_O_5_. Owing to the advantages of high‐pressure/high‐temperature synthesis and sophisticated single‐crystal X‐ray structure analysis, new possibilities in the research of mixed‐valent rare earth oxides arise. The synthesis of high‐pressure mixed‐valent oxides of Ce, Pr, Eu, but also Nd, Sm, Dy, Tm, and Yb, will therefore be an interesting objective for future research.

## Conflict of interest

The authors declare no conflict of interest.

## Supporting information

As a service to our authors and readers, this journal provides supporting information supplied by the authors. Such materials are peer reviewed and may be re‐organized for online delivery, but are not copy‐edited or typeset. Technical support issues arising from supporting information (other than missing files) should be addressed to the authors.

Supplementary
