# Supplementary material for: The High‐Pressure Oxide Tb3O5 and its Non‐Centrosymmetric Low‐Temperature Polymorph–A Comprehensive Study
Source: Chemistry. 2018 Oct 1;24(57):15236–45. doi: 10.1002/chem.201804006 (PMC6220859; doi:10.1002/chem.201804006)
Supplement: Supplementary file 1 — Supplementary [file CHEM-24-15236-s001.pdf]

CHEMISTRY
A European Journal

Supporting Information

The High-Pressure Oxide Tb_3O_5 and its Non-Centrosymmetric Low-Temperature Polymorph–A Comprehensive Study

Matthias Glätzle,[a] Oliver Janka,[b] Volodymyr Svitlyk,[c] Dmitry Chernyshov,[c]
Manfred Bartsch,[d] Helmut Zacharias,[d] Rainer Pöttgen,[b] and Hubert Huppertz*[a]

chem_201804006_sm_miscellaneous_information.pdf

Supporting Information for

Experimental Methods

Synthesis

A polycrystalline sample of HT-HP-Tb_3O_5 was synthesized under high-pressure/high-temperature conditions of 8 GPa and 1050 °C from an equimolar mixture of "Tb_4O_7" (Smart Elements, Vienna, Austria, 99.99%) and Tb_2O_3 (Sigma Aldrich, Vienna, Austria, 99.99%). "Tb_4O_7" alone was found to be applicable as the starting material as well, due to the reducing effect of the α-BN crucible on the reactants. Powder X-ray diffraction analyses of the educts showed no significant crystalline impurities. The educt mixture was finely ground under Ar inert gas atmosphere and filled into boron nitride crucibles (Henze BNP AG, Lauben, Germany), which were sealed with a boron nitride lid. These crucibles were positioned in the center of 18/11 assemblies, which were compressed by eight tungsten carbide cubes (Hawedia, ha-7%Co, Marklkofen, Germany). Details on the construction of the assembly are described in the literature.[1] Quasi-hydrostatic pressure was applied by a Walker-type multianvil module and a 1000 ton press (both Max Voggenreiter GmbH, Mainleus, Germany). Heating was supplied by a resistive graphite tube heating. The sample was compressed up to 8 GPa within 3.5 h, followed by heating it up to 1323 K in 8 min, keeping this temperature for 15 min, and cooling down to 773 K within 20 min. After quenching down to room temperature by switching off the heating, the decompression of the assembly required 10.5 h. The product was isolated by breaking apart the octahedral pressure medium (MgO, Ceramic Substrates & Components Ltd., Newport, Isle of Wight, UK) and careful separation from the surrounding graphite and boron nitride. HT-HP-Tb_3O_5 was obtained as an air- and water-resistant crystalline product. The color of the product sample changed from dark grey to reddish brown – typical for a mixed-valent compound due to electron charge transfer between Tb^{3+} and Tb^{4+} – when grinding for the purpose of further investigation.

Results and Discussion

Crystal structure of HT-HP-Tb_3O_5

As reported in the main article, the Tb1 ions are coordinated by eight oxygen anions with interatomic distances between 233 and 253 pm in form of a bi-capped trigonal prism. However, another oxygen atom at a distance of 332 pm caps the third face of the trigonal prism, therefore forming a tri-capped trigonal prismatic coordination of Tb1 (see Figure S1). For HT-HP-Tb_3O_5, this oxygen anion cannot be regarded coordinating Tb1 due to its large interatomic distance, though it is included in the coordination description of atoms at the Sb2 position of some intermetallics crystallizing in the β-Yb_5Sb_3 type structure, e.g. Ti_5Sb_3.[2] This is due to the smaller relative interatomic distance deviation of the three capping atoms in Ti_5Sb_3 (335, 334, and 362 pm) in comparison to Tb_3O_5 (240, 233, and 332 pm). Similarly, the second crystallographic independent anions on the Sb1 position are ascribed to be coordinated by eight cations in form of a bi-capped distorted octahedron rather than seven as in HT-HP-Tb_3O_5 (Figure S1). Furthermore, some of the square pyramids are transformed into octahedra by the approach of a sixth atom and the tetrahedra change into trigonal bipyramids in a similar way in for instance Ti_5Sb_3.[2]

The elongated hexagons formed by the oxygen anions as the base planes of the hexagonal prisms in the crystal structure of HT-HP-Tb_3O_5 form a parquet-like patchwork, as is shown in Figure S3. This pattern is also found, more or less distorted, in the structure of Rh_5Ge_3 and all its derivative structure types.[3] We should note that this is the typical symmetry imposed by space group *Pnma*.

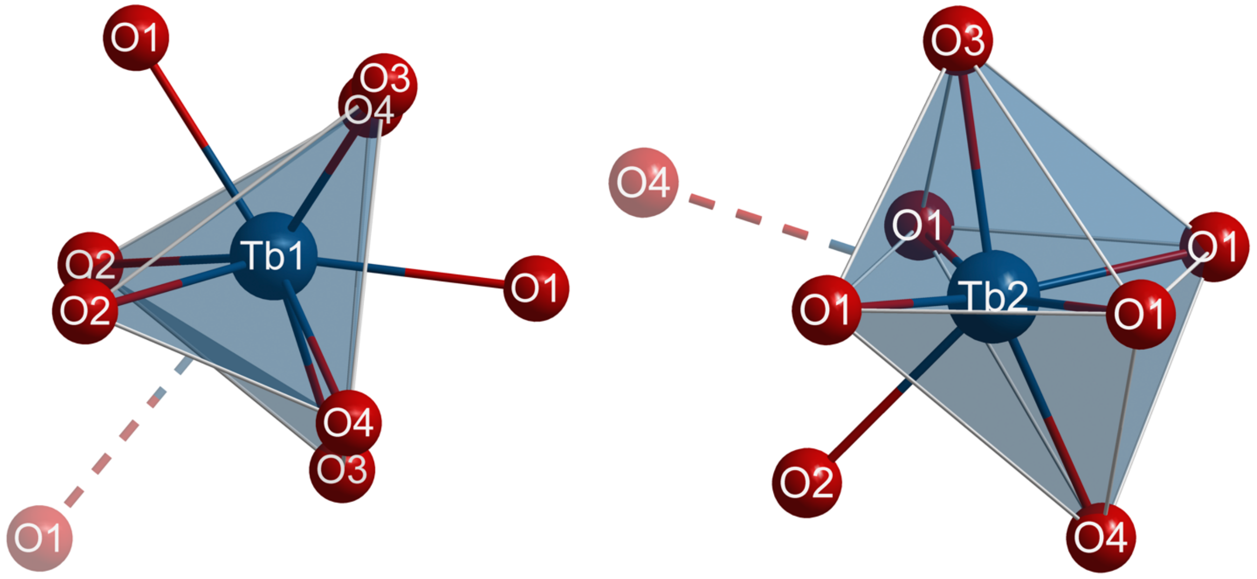

Figure S1. Coordination of the terbium atoms in the crystal structure of HT-HP-Tb_3O_5, including one more non-coordinating oxygen atom each for comparison with isostructural compounds.

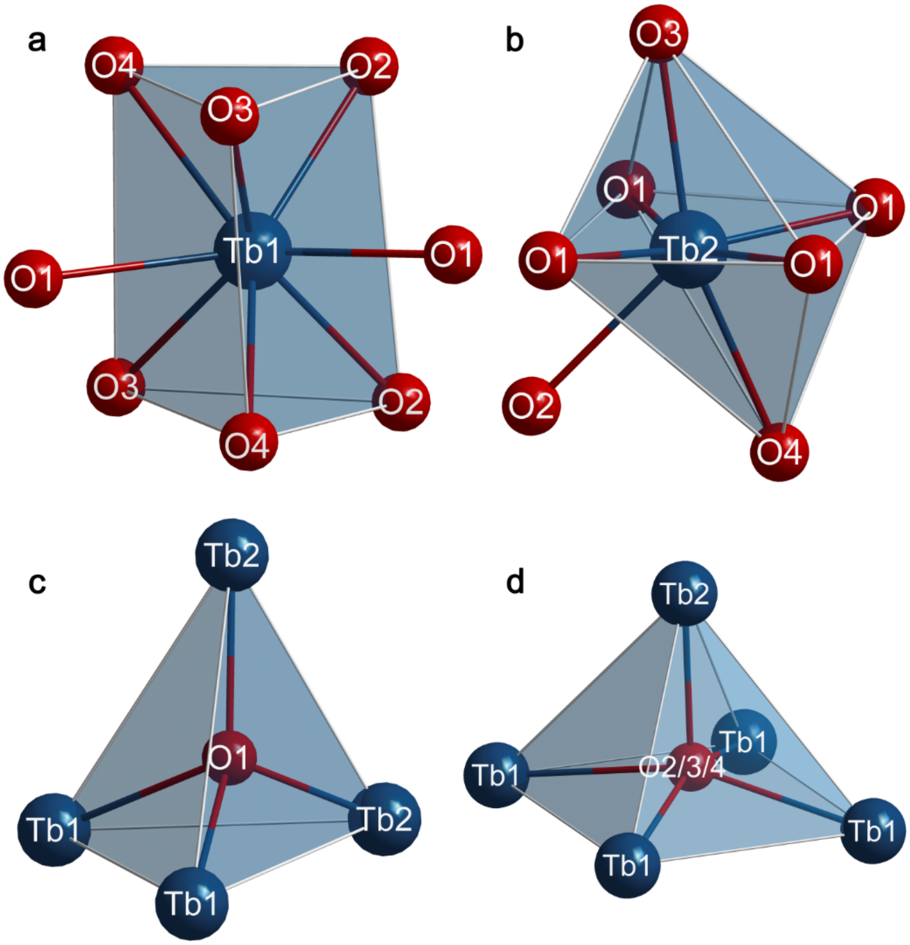

Figure S2. Top: Coordination of the terbium atoms in the crystal structure of HT-HP-Tb_3O_5, highlighting the bi-capped trigonal prismatic coordination of Tb1 (a) and the mono-capped distorted octahedral coordination of Tb2 (b). Bottom: Coordination of the oxygen atoms O1 (c) and O2,3,4 (d) in the crystal structure of HT-HP-Tb_3O_5.

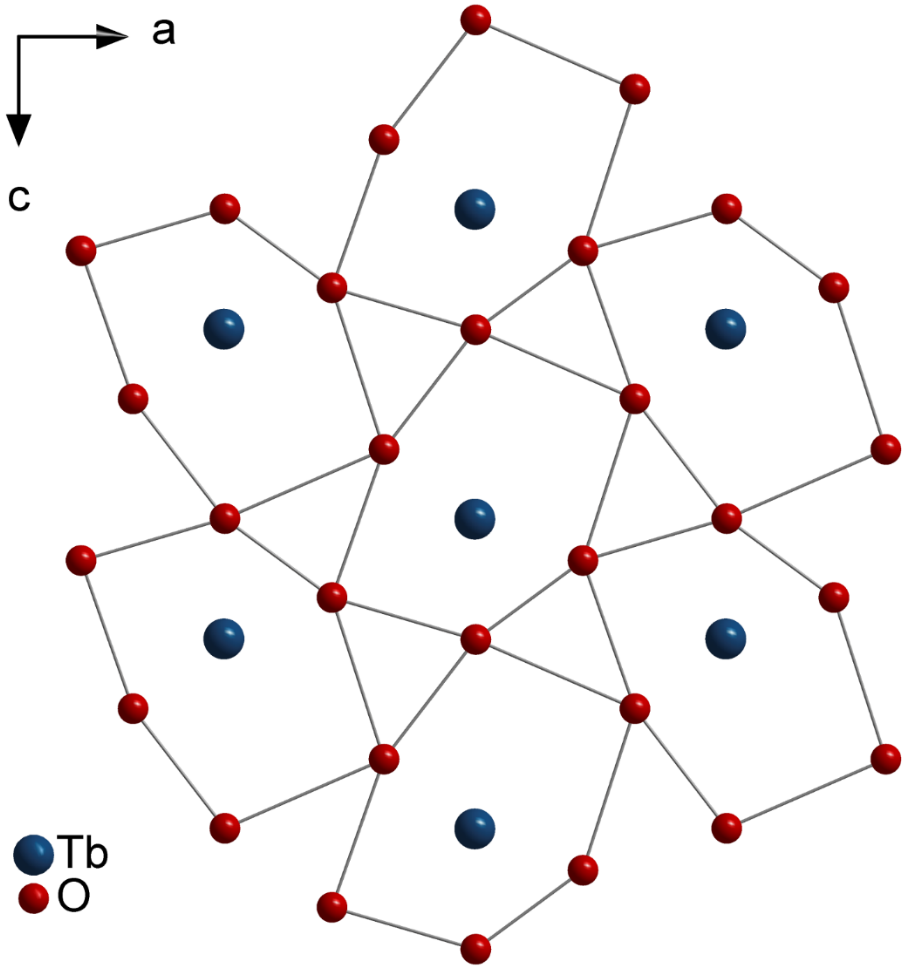

Figure S3. Layer of atoms in HT-HP-Tb_3O_5 at y = ¼ as viewed along [010], highlighting the elongated hexagons built by the oxygen atoms and the terbium atoms off center of these hexagons. The nature of the parquet-like pattern of these hexagons is characteristic for compounds exhibiting the β-Yb_5Sb_3 type structure (in general for space group *Pnma*).

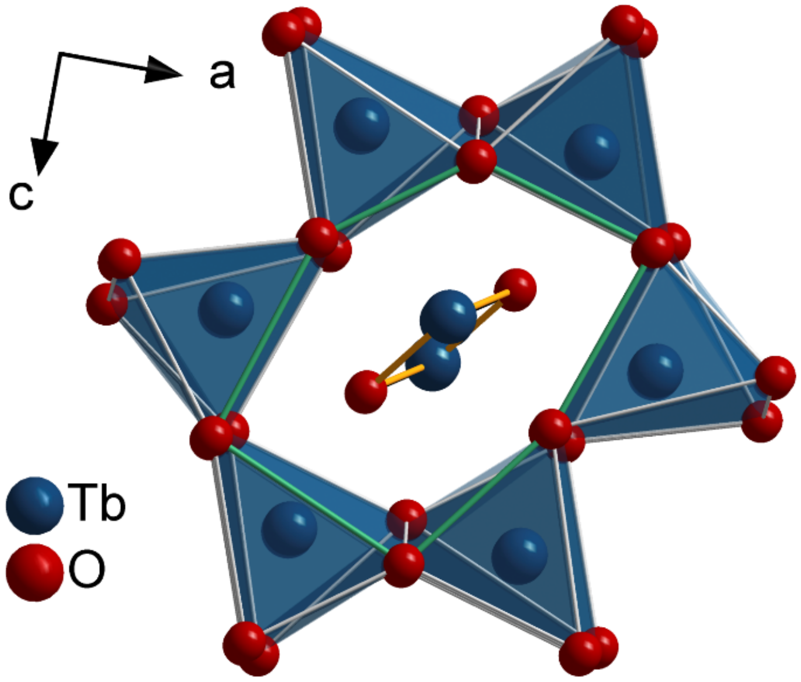

Figure S4. Hexagonal channels in the crystal structure of HT-HP-Tb_3O_5 viewed along [010], highlighting the hexagonal base plane (green) and the zig-zag chain of rhombs in the centre of the hexagonal channels.

Table S1. Powder and single-crystal X-ray diffraction data and structural parameters from refinement of HT-HP-Tb_3O_5 (standard deviations in parentheses).

Empirical formula	Tb_3O_5
Formula weight	556.76
Crystal system	orthorhombic
Space group	*Pnma*
Crystal color	red brown
**Powder data**	
Powder diffractometer	Stoe Stadi P
Radiation; λ, pm	Mo-$K_{\alpha 1}$; 70.93
a, pm	978.97(8)
b, pm	670.36(4)
c, pm	604.35(4)
V, nm³	0.39661(3)
**Single-crystal data**	
Single-crystal diffractometer	Bruker D8 Quest (Photon 100)
Radiation; λ, pm	Mo-K_{α}; 71.073
a, pm	979.30(6)
b, pm	670.64(4)
c, pm	604.23(3)
V, nm³	0.39683(4)
Formula units per cell (Z)	4
Calculated density, g cm^{-3}	9.32
Crystal size, mm³	0.06 × 0.04 × 0.04
Temperature, K	300(2)
Absorption coefficient, mm^{-1}	52.8
F(000), e	940
θ range, deg	3.7 35.0
Range in *hkl*	±15, ±10, ±9
Total no. of reflections	20919
Independent reflections / R_{int}	939 / 0.0378
Reflections with I > $2\sigma(I)$ / R_{σ}	893 / 0.0149
Data / ref. parameters	939 / 44
Absorption correction	multi-scan (Bruker Difabs 2014/5)[4]
GoF on F^2	1.202
R_1 / wR_2 [$I > 2\sigma(I)$]	0.0150 / 0.0366
R_1 / wR_2 (all data)	0.0160 / 0.0371
Residual density, × 10^{-6} e pm^{-3}	2.55 / -1.19

Table S2. Synchrotron powder X-ray diffraction data and structure refinements for HT-HP-Tb_3O_5 (300 K) and LT-HP-Tb_3O_5 (2 K).

Compound		HT-HP-Tb_3O_5	LT-HP-Tb_3O_5
Temperature		300 K	2 K
Space group		*Pnma*	*Pna*2_1
Unit cell dimensions	a =	979.00(1)	978.53(1)
(in pm)	b =	670.48(1)	603.23(1)
	c =	604.34(1)	668.45(1)
Volume, nm^3	V =	0.3967	0.3946
Wavelength, nm		0.6979	0.6979
θ range, °		0.10-51.19	0.07-34.63
Data points / increment		3352 / 0.015	3352 / 0.010
Number of reflections		455	152
Number of parameters[a]		26	28
R-factor / *wR*-factor		2.03 / 2.91	2.54 / 4.21

[a] last run.

Table S3. Atomic coordinates and isotropic equivalent displacement parameters (U_{eq} in Å²) for HT-HP-Tb_3O_5 (space group *Pnma*) based on single-crystal X-ray diffraction data. U_{eq} is defined as one third of the trace of the orthogonalized U_{ij} tensor.

Atom	Wyckoff position	x	y	z	U_{eq}
Tb1	8*d*	0.17400(2)	0.00182(2)	0.51791(2)	0.00475(5)
Tb2	*4c*	0.00538(2)	¼	0.05668(3)	0.00381(5)
O1	8*d*	0.0970(2)	0.5283(3)	0.1545(3)	0.0059(3)
O2	4*c*	0.0034(2)	¼	0.4452(5)	0.0066(5)
O3	4*c*	0.1863(3)	¼	0.8315(5)	0.0065(5)
O4	4*c*	0.2899(3)	¼	0.3098(5)	0.0059 (5)

Table S4. Atomic coordinates and isotropic equivalent displacement parameters (U_{eq} in pm²) for HT-HP-Tb_3O_5 (*Pnma*, 300 K) and LT-HP-Tb_3O_5 (*Pna*2$_1$, 2 K) synchrotron powder X-ray diffraction data. U_{eq} is defined as one third of the trace of the orthogonalized U_{ij} tensor.

Atom	*Wyckoff* position	*x*	*y*	*z*	U_{eq}
HT-HP-Tb_3O_5					
Tb1	8*d*	0.17308(5)	0.0006(3)	0.51774(10)	35(1)
Tb2	4*c*	0.00306(19)	1/4	0.05746(11)	15(2)
O1	8*d*	0.1036(9)	0.5391(16)	0.1354(15)	168(1)
O2	4*c*	0.013(2)	1/4	0.4093(16)	168(1)^{a}
O3	4*c*	0.1940(16)	1/4	0.8559(19)	168(1)^{a}
O4	4*c*	0.2857(15)	1/4	0.306(2)	168(1)^{a}
LT-HP-Tb_3O_5					
Tb1	4*a*	0.1763(5)	0.5193(8)	0.9983(10)	38^{c}
Tb2	4*a*	0.0056(4)	0.0579(12)	0.75^{b}	38^{c}
Tb3	4*a*	0.8277(5)	0.4862(4)	0.0038(3)	38^{c}
O1	4*a*	0.107(4)	0.148(5)	0.490(5)	127^{c}
O2	4*a*	0.003(3)	0.441(4)	0.705(3)	127^{c}
O3	4*a*	0.193(4)	0.861(13)	0.761(3)	127^{c}
O4	4*a*	0.287(6)	0.321(9)	0.732(4)	127^{c}
O5	4*a*	0.911(3)	0.854(4)	0.579(6)	127^{c}

^{a} U_{eq} of O1 refined, U_{eq}'s of O2, O3 and O4 fixed equal to O1; ^{b} parameter fixed due to floating origin in space group *Pna*2$_1$; ^{c} U_{eq}'s for all atoms fixed in last run.

Table S5. Anisotropic equivalent displacement parameters U_{ij} (Å²) for HT-HP-Tb_3O_5 (space group *Pnma*) based on single-crystal X-ray diffraction data.

Atom	U_{11}	U_{22}	U_{33}	U_{23}	U_{13}	U_{12}
Tb1	0.00396(7)	0.00517(7)	0.00511(8)	-0.00085(4)	0.00028(3)	-0.00013(3)
Tb2	0.00317(8)	0.00358(8)	0.00467(9)	0	0.00032(5)	0
O1	0.0063(8)	0.0063(7)	0.0052(8)	0.0004(6)	-0.0003(6)	-0.0004(6)
O2	0.003(2)	0.009(2)	0.007(2)	0	0.000(1)	0
O3	0.006(1)	0.007(1)	0.007(2)	0	0.0022(9)	0
O4	0.005(1)	0.006(1)	0.007(2)	0	0.000(1)	0

Table S6. Interatomic distances (given in pm) for HT-HP-Tb_3O_5 (*Pnma*, single-crystal data) and LT-HP-Tb_3O_5 (*Pna*2_1, powder data). Standard deviations for the single-crystal data are ±0.2 pm, for the powder diffraction data ±1 pm.

HT-HP-Tb_3O_5				LT-HP-Tb_3O_5					
Tb1		O1	233.0	Tb1	O2	225	Tb3	O2	218
		O4	237.5		O1	226		O4	222
		O1	239.6		O3	237		O3	229
		O2	239.9		O4	240		O1	230
		O2	243.3		O4	243		O5	252
		O3	244.8		O5	247		O4	263
		O4	246.7		O3	261		O2	265
		O2	252.5		O2	264		O3	272
		∅	**242.2**		∅	**243**		∅	**244**
Tb2	2×	O1	215.4	Tb2	O5	192			
	2×	O1	220.1		O1	207			
		O3	223.4		O3	219			
		O4	225.9		O4	226			
		O2	234.7		O1	231			
		∅	**222.1**		O2	233			
					O5	241			
					∅	**221**			
O1		Tb2	215.4	O1	Tb2	207	O5	Tb2	192
		Tb2	220.1		Tb1	226		Tb2	241
		Tb1	233.0		Tb3	230		Tb1	247
		Tb1	239.6		Tb2	231		Tb3	252
		∅	**227.0**		∅	**224**		∅	**233**
O2		Tb2	234.7	O2	Tb3	218			
	2×	Tb1	239.9		Tb1	225			
	2×	Tb1	243.3		Tb2	233			
		∅	**240.2**		Tb1	264			
					Tb3	265			
					∅	**241**			
O3		Tb2	223.4	O3	Tb2	219			
	2×	Tb1	244.8		Tb3	229			
	2×	Tb1	252.5		Tb1	237			
		∅	**243.6**		Tb1	261			
					Tb3	272			
					∅	**244**			
O4		Tb2	225.9	O4	Tb3	222			
	2×	Tb1	237.5		Tb2	226			
	2×	Tb1	246.7		Tb1	240			
		∅	**238.9**		Tb1	243			
					Tb3	263			
					∅	**239**			

Calculations of the Madelung part of lattice energy (MAPLE)

The MAPLE[5] values for HT-HP-Tb_3O_5 and LT-HP-Tb_3O_5 were calculated based on the crystal structures and compared with the values received from the oxides used as starting material. A value of 26,781 kJ mol^{-1} was obtained for HT-HP-Tb_3O_5, to be compared with 27,132 kJ mol^{-1} (deviation: 1.29%) resulting from the starting materials [1/3 · Tb_2O_3[6] (15,054 kJ mol^{-1}) + 1/3 · Tb_7O_{12}[7] (66,341 kJ mol^{-1})]. For LT-HP-Tb_3O_5, a value of 26,928 kJ mol^{-1} was obtained, to be compared with 27,132 kJ mol^{-1} (deviation: 0.75%). This validates the accuracy of the crystal structure solution of HT-HP-Tb_3O_5 and LT-HP-Tb_3O_5.

Further details on the charge distribution calculations

Comprehensive calculations and discussions regarding the valence state of the rare earth cations in various cerium oxides – namely the *A*- and *C*-type sesquioxides, the dioxide and some intermediate higher oxides – based on calculations applying the bond valence model (*BLBS*) are reported by Shoko et al.[8] They concluded that Ce_3O_5 is a homogeneous mixed-valence compound with comparable valences of 3.33 at the Ce1 and 3.37 at the Ce2 sites. This can be argued by the comparable polyhedron sizes with almost equal average Ce–O distances of 244 pm. However, the average Tb–O distances in HT-HP-Tb_3O_5 are 242 pm for Tb1 and 222 pm for Tb2 (see Table S6). This huge difference in mean interatomic distances accounts for the clearly unequal valence of the two terbium sites in HT-HP-Tb_3O_5, which could therefore be ascribed Class 1 according to Robin and Day[9] with the two distinct crystallographic sites having integral but unequal valence. Nevertheless, the results obtained by the *CHARDI* calculations, the lower coordination number of Tb2 of seven, and the fact that the HT-HP-Tb_3O_5 samples obviously show a strong optical absorption in the visible spectrum rather speak for a Class 2 inhomogeneous mixed valence of the terbium cations.

Powder X-ray diffraction

The experimental powder X-ray diffraction pattern of the reaction product (Figure S5) is consistent with the theoretical powder pattern simulated from single-crystal X-ray diffraction data. Thus, the experimental powder pattern shows some additional reflections (marked with asterisks) assignable to Tb_6O_{11} arising as a side product. By indexing the reflections of HT-HP-Tb_3O_5, we obtained the parameters a = 978.97(8), b = 670.36(4), c = 604.35(4) pm, and a volume of 0.39661(3) nm^3.

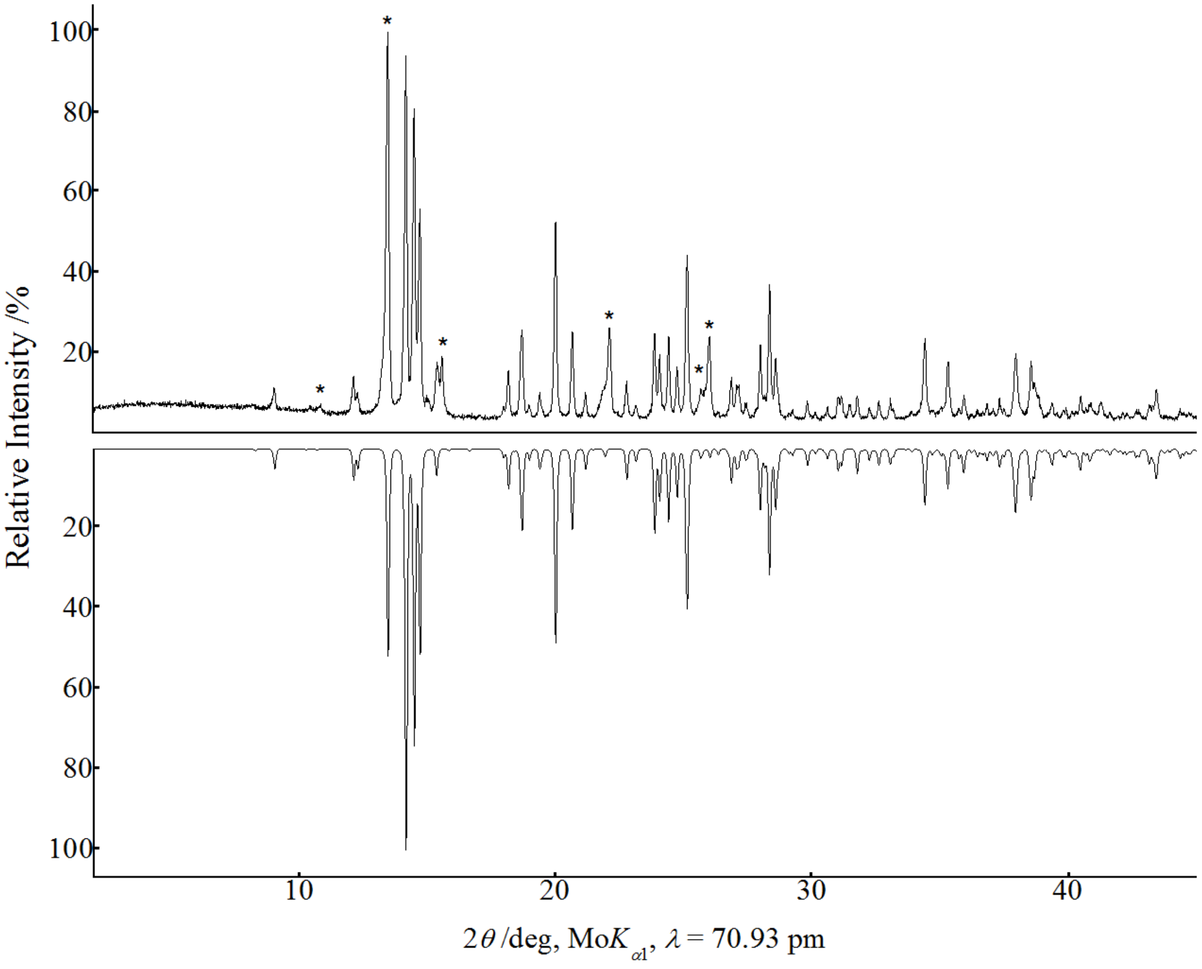

Figure S5: Experimental powder X-ray diffraction pattern of HT-HP-Tb_3O_5 (top) in comparison to the theoretical powder pattern simulated from single-crystal X-ray diffraction data (bottom). Reflections marked with asterisks originate from Tb_6O_{11}.

References

[1] a) D. Walker, M. A. Carpenter, C. M. Hitch, *Am. Mineral.* **1990**, *75*, 1020-1028; b) D. Walker, *Am. Mineral.* **1991**, *76*, 1092-1100; c) H. Huppertz, *Z. Kristallogr. - Cryst. Mater.* **2004**, *219*, 330-338.

[2] R. Berger, *Acta Chem. Scand., Ser. A* **1977**, *31*, 889-890.

[3] R. E. Gladyshevskii, K. Cenzual, J. T. Zhao, E. Parthé, *Acta Crystallogr., Sect. C: Cryst. Struct. Commun.* **1992**, *48*, 221-225.

[4] a) G. M. Sheldrick, *SADABS* v. 2014/5, Bruker/Siemens Area Detector Absorption Correction Program, Bruker AXS Inc., Madison, Wisconsin, USA, **2014**; b) Bruker, *SAINT* v. 8.34a, Bruker AXS Inc., Madison, Wisconsin, USA, **2013**.

[5] a) R. Hoppe, *Angew. Chem., Int. Ed. Engl.* **1966**, *5*, 95-106; b) R. Hoppe, *Angew. Chem., Int. Ed. Engl.* **1970**, *9*, 25-34; c) R. Hübenthal, Maple, Program for the Calculation of MAPLE Values, University of Gießen, Gießen, Germany, **1993**.

[6] E. Hubbert-Paletta, H. Müller-Buschbaum, *Z. Anorg. Allg. Chem.* **1968**, *363*, 145-150.

[7] J. Zhang, R. B. Von Dreele, L. Eyring, *J. Solid State Chem.* **1993**, *104*, 21-32.

[8] a) E. Shoko, M. F. Smith, R. H. McKenzie, *J. Phys.: Condens. Matter* **2010**, *22*, 223201; b) E. Shoko, M. F. Smith, R. H. McKenzie, *J. Phys. Chem. Solids* **2011**, *72*, 1482-1494; c) E. Shoko, M. F. Smith, R. H. McKenzie, *Phys. Rev. B: Condens. Matter Mater. Phys.* **2009**, *79*, 134108.

[9] M. B. Robin, P. Day in *Molecules Into Materials: Case Studies in Materials Chemistry – Mixed Valency, Magnetism and Superconductivity*, World Scientific, **2007**, pp. 142-317.
